# Safety and caregiver satisfaction with gastrostomy in patients with Ataxia Telangiectasia

**DOI:** 10.1186/1750-1172-6-23

**Published:** 2011-05-15

**Authors:** Maureen A Lefton-Greif, Thomas O Crawford, Sharon McGrath-Morrow, Kathryn A Carson, Howard M Lederman

**Affiliations:** 1 The Ataxia Telangiectasia Clinical Center, Johns Hopkins Medical Institutions, Baltimore, Maryland. USA; 2 The Eudowood Division of Pediatric Respiratory Sciences, Johns Hopkins Medical Institutions, Baltimore, Maryland. USA; 3 Department of Pediatrics, Johns Hopkins Medical Institutions, Baltimore, Maryland. USA; 4 Department of Neurology, Johns Hopkins Medical Institutions, Baltimore, Maryland. USA; 5 Department of Epidemiology, Johns Hopkins Bloomberg School of Public Health, Baltimore, Maryland, USA; 6 The Eudowood Division of Pediatric Allergy and Immunology, Johns Hopkins Medical Institutions, Baltimore, Maryland. USA

## Abstract

**Background:**

Ataxia Telangiectasia (A-T) is a rare monogenetic neurodegenerative disease with pulmonary, nutritional, and dysphagic complications. Gastrostomy tube (GT) feedings are commonly recommended to manage these co-morbidities. In general, outcomes of GT placement in patients with progressive diseases that develop during childhood are not well characterized. The primary purposes of this study were to determine whether GT placement in patients with A-T would be tolerated and associated with caregiver satisfaction.

**Methods:**

We completed a retrospective review of 175 patients who visited the A-T Children's Center at Johns Hopkins Hospital from 2001 through 2008, and identified 28 patients with A-T (19 males, 9 females) who underwent GT placement for non-palliative reasons. Information was obtained from medical records, interviews with primary health care providers, and 24 (83%) caregivers of patients with GT's who responded to survey requests.

**Results:**

Twenty-five (89%) patients tolerated GT placement and were a median of 5.0 (0.4-12.6) years post GT placement at the time of this investigation. Three (11%) patients died within one month of GT placement. In comparison to patients who tolerated GT placement, patients with early mortality were older when GT's were placed (median 24.9 vs. 12.3 years, p = 0.006) and had developed a combination of dysphagia, nutritional, and respiratory problems. Caregivers of patients tolerating GT placement reported significant improvements in mealtime satisfaction and participation in daily activities.

**Conclusions:**

GT placement can be well tolerated and associated with easier mealtimes in patients with A-T when feeding tubes are placed at young ages. Patients with childhood onset of disorders with predictable progression of the disease process and impaired swallowing may benefit from early versus late placement of feeding tubes.

## Background

Ataxia Telangiectasia (A-T) is a rare neurodegenerative disease characterized by ataxia, immunodeficiency, sinopulmonary infections, premature aging, nutritional compromise and oropharyngeal dysphagia[1-6]. The prevalence of A-T is estimated to be between 1 in 40,000 and 1 in 300,000 live births[7-9]. Life expectancy has increased from 19 to 25 years, with respiratory failure, complications of chemotherapy or cancer, and neurologic deterioration remaining the leading causes of death[10]. Increased morbidity and mortality is associated with impairments in deglutition, nutritional status, immune function, and neurologic status in other chronic progressive conditions [11-14]. Treatments that improve nutritional status and minimize the risk of aspiration for patients with chronic progressive disorders are salutary[15-20]. Thus it is important to provide information that guides clinicians in the selection of interventions for nutrition and aspiration induced lung injury with progressive conditions.

Gastrostomy tube (GT) feedings are commonly used to manage dysphagia with concomitant aspiration, or manage chronic conditions associated with nutritional compromise that are refractory to less aggressive adjustments in feeding routine[18,21-23]. Nonetheless, outcomes associated with GT placement are mixed. Whereas some investigators report that GT placement leads to significant improvements in growth and health parameters and caregivers' perceptions,[2,15,18,24-27] others have cautioned about adverse outcomes and questioned whether available evidence supports the cost and effectiveness of GT[28-30]. In our experience, this controversy frequently results in postponing the placement of feeding tubes, particularly when GT placement is perceived as a measure of last resort.

The national clinical center for A-T at the Johns Hopkins Clinical Center (ATCC) attracts a large cohort of patients and afforded the opportunity to identify clinical characteristics associated with outcome following GT placement. We hypothesized that GT placement would be well tolerated and viewed positively by both caregivers and patients with A-T. Our secondary goals were to elucidate clinical characteristics of patients who would benefit from feeding tubes and improve our understanding of when to recommend placement of feeding tubes.

## Methods

Between October 2001 and September 2008, 175 patients with A-T were evaluated at the Johns Hopkins ATCC. Criteria for a diagnosis of A-T included the presence of characteristic neurologic features (gait ataxia, oculomotor dysfunction, dysarthria, and a movement disorder) and at least one of the following: oculocutaneous telangiectasia, elevated serum levels of alpha-fetoprotein, or spontaneous or x-irradiation-induced chromosomal breakage[31]. We identified patients who had GT's placed before or after clinic visits to our center. The A-T Children's Project (an organization dedicated to patient support and research) identified additional patients who had GT's placed after their visits to our center. Between July 2005 and September 2008, the nurse coordinator at the ATCC contacted caregivers of all patients who were identified as having GT's placed before May 2008. Each caregiver was asked to complete a survey on caregiver and patient satisfaction pre- and post-GT placement. (Additional File 1) The nurse coordinator followed up with phone calls to caregivers and read survey questions to any caregivers who requested help to complete the survey. Demographic information, pre-GT BMI Z-scores, neurologic scores, dysphagia and immunologic status, reason for GT placement, and cause of death information were obtained from medical records and follow up calls to the offices of primary health care providers. Survival data were collected through September 2008, 4 months or longer after GT placement. Pre-GT BMI Z-scores were obtained less than two years before GT placement. Neurologic scores were calculated from the Quantitative Neurologic Assessment of Ataxia Telangiectasia index within two years of GT placement[4]. Lower neurologic scores are associated with greater disease progression. Dysphagic problems were determined by clinical presentations and videofluoroscopic swallow study examinations as previously described[3]. The protocol for this study was approved by the Institutional Review Board of The Johns Hopkins Medical Institution.

### Statistical Analysis

Patients were categorized as tolerating GT placement if they survived 30 or more days following GT placement. Patients who survived less than 30 days after GT placement are henceforth referred to as patients with early mortality. Fisher's exact tests and Wilcoxon rank sum tests were used to compare patient characteristics of those who tolerated GT placement and those with early mortality. Pearson correlation was used to assess the association of neurologic score and age at time of GT placement. Because of the limited sample size, we used univariable logistic regression analysis with Firth's bias correction for continuous measures and exact methods for categorical measures to determine factors associated with tolerating GT placement. Pre- and post-GT BMI z-scores were compared using Wilcoxon signed rank test. Mealtime satisfaction and energy to participate in daily activities pre- and post-GT placement were compared using paired *t*-tests. Statistical analysis was performed using SAS version 9.2 (SAS Institute, Inc., Cary, NC). All *P *values reported are two-sided and statistical significance was set at *p *< 0.05.

## Results

**Figure 1:**
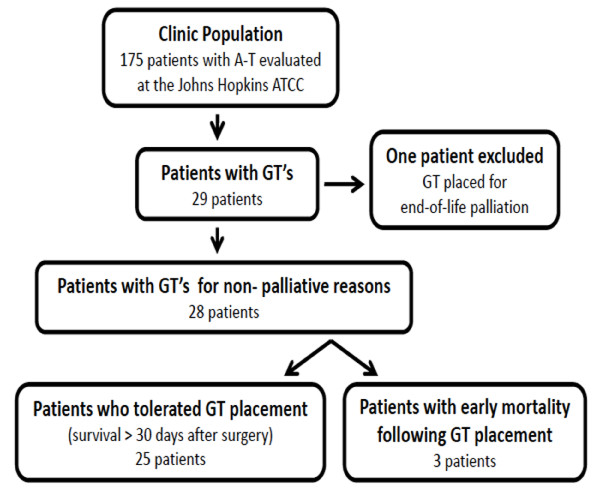
**Population derivations**.

Twenty-nine patients with A-T were identified from our outreach and chart review as having undergone GT placement (Figure 1). Chart reviews were completed on average 4.11 years (median, range: 3.58, 0.28-11.16 years) after GT placement. One of these patients underwent GT placement specifically for palliative care and was excluded from the analysis. Individual patient characteristics for the 28 patients included are described in Table 1. The median (range) age at the time of GT placement was 12.6 years (4.6-27.0 years), 19 (68%) were male and 9 (32%) were female. All patients had GT placed for nutrition support and 12 (43%) also had dysphagia.

**Table 1:** Characteristics of patients tolerating gastrostomy tube (GT) placement and patients with early mortality following GT placement

Pt.	Sex	Age GT Placed(yrs)	Years Post GT	BMI Z-Score^1^	A-T Neurologic Score^2^	Dysphagia^3^	Immunologic Status		Reason(s) for GT	Alive at Time of Survey	Cause of Death
										
							IgA	IgG			
Patients tolerating GT placement											

1	F	10	<1			No	Normal	Deficient	Nutrition	Yes	
2	M	15	<1	-1.76	35	Yes: P	Deficient	Normal	Nutrition, GER treated w/nissen fundoplication	Yes	
3	M	6	1	0.90		No	Normal	Deficient	Nutrition, GER, pulmonary infections	Yes	
4	F	10	1	-6.23	68	No	Normal	Deficient	Nutrition	Yes	
5	M	12	1	-2.77	51	No	Deficient	Normal	Nutrition	Yes	
6	F	13	1			Yes: P	Normal	Normal	Nutrition, dysphagia	Yes	
7	F	15	1	-0.44	40	No	Deficient	Deficient	Nutrition	Yes	
8	M	15	1	-5.72	36	No	Normal	Normal	Nutrition, dysphagia	Yes	
9	M	10	2	-8.17	59	No	Deficient	Normal	Nutrition	Yes	
10	M	10	3			No	Deficient	Normal	Nutrition, cancer	Yes	
11	M	13	3	-0.65	38	Yes: P	Deficient	Normal	Nutrition, LL pneumonia	Yes	
12	M	10	4	-4.25	32	Yes: A	Deficient	Normal	Nutrition, dysphagia	Yes	
13	M	14	4	-4.42	22	Yes: A	Deficient	Normal	Nutrition, dysphagia	Yes	
14	M	16	4		41	Yes: A	Deficient	Normal	Nutrition, dysphagia	No	Leukemia, lung complications
15	M	10	5	-0.79	47	Yes: A	Deficient	Deficient	Nutrition, dysphagia	No	Burkitt lymphoma; lung complications; recurrent aspiration
16	F	12	5	-7.57	39	Yes: P	Deficient	Normal	Nutrition, dysphagia	No	Lung complications, failure to thrive, recurrent aspiration
17	M	13	5	-3.39	53	Yes: P	Deficient	Deficient	Nutrition, dysphagia	Yes	
18	M	15	6			No	Deficient	Normal	Nutrition	No	Non-Hodgkin Lymphoma T cell
19	M	17	6			Yes: A, R	Deficient	Normal	Nutrition	No	Aspiration pneumonia
20	M	5	8			No	Normal	Deficient		No	Hodgkin lymphoma
21	F	11	8	-3.12	35	No	Normal	Normal	Nutrition	Yes	
22	M	17	8	-5.03	21	Yes: P, R	Normal	Normal	Nutrition, dysphagia	No	T-Cell Lymphoma, lung complications
23	M	7	9			No	Deficient	Normal	Nutrition	Yes	
24	M	15	9	-11.89	19	Yes: P	Deficient	Deficient		No	Congestive heart failure
25	F	6	11			No	Deficient	Normal	Nutrition, recurrent lung infections	No	Congestive heart failure, lung disease

Patients with early mortality following GT placement											

1	F	22	0	-7.03	50	Yes: P, R	Normal	Normal	Nutrition (10 lb weight loss/3 yrs), dysphagia, recurrent pneumonia	No	Did not tolerate GT feedings, pneumonia, and respiratory failure,
2	F	25	0	-1.10	53	Yes: P	Not Done	Not Done	Nutrition (10 lb weight loss/1 yr), dysphagia, chronic cough, declining respiratory function (FVC = 20% predicted)	No	Did not tolerate GT feedings, pneumothorax, and respiratory failure
3	M	27	0	-7.78	15	Yes: P, R	Deficient	Normal	Nutrition (16 lb weight loss/1 yr), dysphagia	No	Did not tolerate GT feedings, pneumonia, pneumothorax, and respiratory failure

^1^BMI Z-scores were obtained less than 2 years before GT placement.

^2^A-T neurologic scores were obtained less than 2 years before or after GT placement

^3^Dysphagia was defined as Videofluoroscopic Swallow Study findings of Penetration (P), Aspiration (A) or Post-Swallow Residue (R).

Abbr: BMI, body mass index

Twenty-five (89%) patients survived more than 30 days after the procedure. Patients tolerating GT placement had feeding tubes placed at a median (range) age of 12.3 (4.6-17.0) years and at the time of our retrospective assessment were 5.0 (0.4-12.6) years post tube placement.

At the time of this investigation, 16 (64%) of patients tolerating GT placement, were alive. The mean age of death was 19.5 ± 4.3 years for those who tolerated GT placement but were deceased at the time of this investigation. Respiratory problems were the leading cause of death and occurred in 6 (67%) of these patients; five (56%) of the deaths were attributed to cancer or other non-respiratory conditions. (Table 1) At the time of data collection, median post GT survival times were marginally shorter for those who tolerated GT placement and were alive versus deceased (4.3 [0.4-12.6] *vs*. 6.3 [4.5-11.1] years, p = 0.05).

Three (11%) patients died within one month of GT insertion. In comparison to patients who tolerated GT insertion, these patients were significantly older when GT's were placed. (Table 2) Although there were an insufficient number of patients to demonstrate statistical significance, each had significant weight loss, dysphagia, and severe respiratory problems. Two patients with early mortality lost more than 10 pounds during the year preceding GT placement and one of these patients had an FVC of 20% predicted. (Table 1)

**Table 2:** Demographic and clinical characteristics for patients tolerating gastrostomy tube (GT) placement and patients with early mortality after GT placement

Characteristics	Patients tolerating GT placement (n = 25)	Patients with early mortality (n = 3)	*P *value*
Male sex, N (%)	18 (72)	1 (33)	0.23
Age at GT placement, years, median (range)	12.3 (4.6-17.0)	24.9 (22.1-27.0)	0.006
Neurologic score, median (range)	41 (19-77)	50 (15-53)	0.55
Body mass index for age, median (range)	-3.82 (-11.89-0.90)^a^	-7.03 (-7.78- -1.10)	0.47
IgA deficient, N (%)	17 (68)	0 (0)	0.13
IgG deficient, N (%)	8 (32)	0 (0)	1.0
Dysphagia, N (%)	12 (48)	3 (100)	0.23

* P value from Fisher's exact test or Wilcoxon rank sum test.

^a ^Data only available on 16 of the 25 patients.

**Figure 2:**
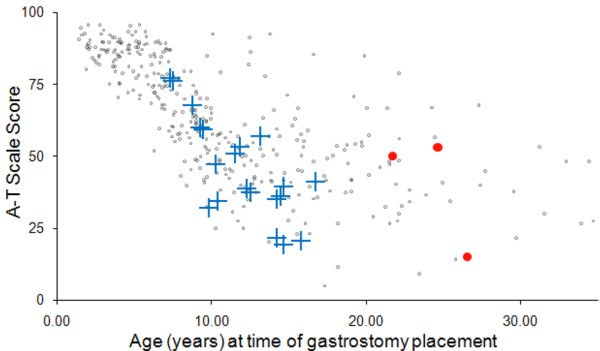
**A-T neurologic score and age of GT placement**. Gray dots represent A-T neurologic scores for all 347 patients with A-T who were evaluated at the ATCC. Blue crosses represent the A-T patients who tolerated GT placement. Red circles represent the three patients with early mortality.

**Figure 3:**
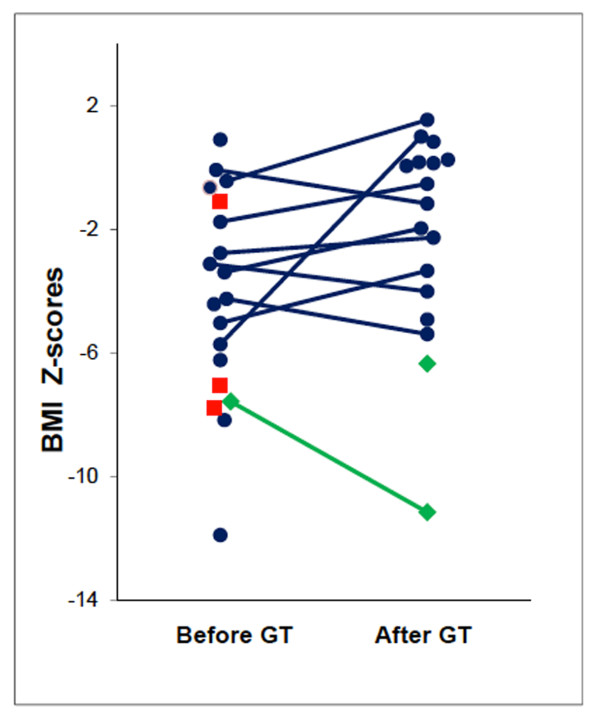
**BMI Z-scores before and after GT placement**. Blue circles represent individual patients who tolerated GT placement and used GT's. Green diamonds represent the two patients who tolerated GT placement but did not use their feeding tubes. Red squares represent the three patients with early mortality. Percentile BMI Z-score is based on NHANES normal values for age and gender. For patients older than 20 years, percentile BMI Z-scores were based upon NHANES normal values for the age of 19 years 11 months and gender. Source for BMI calculations: http://statcoder.com/

Age of GT placement was marginally correlated with neurologic score (Pearson's r = -0.33; p = 0.08). (Figure 2) Age of GT placement was associated with tolerating GT placement (OR = 0.62; 95% confidence interval (CI) = 0.41-0.94); for each year increase in age there was a 38% decrease in the odds of tolerating GT placement. Factors examined that were not statistically significant for association with tolerating GT placement were male gender (OR = 4.81; 95% CI = 0.22-319.8), dysphagia (OR = 0.27; 95% CI = 0.00-2.69), neurologic score (OR = 1.02; 95% CI = 0.96-1.08 for a 1 unit increase) and BMI Z-score (OR = 1.11; 95% CI = 0.78-1.57 for a 1 unit increase). (Table 3) Pre-GT BMI Z-scores were obtained less than two years (median: 0.50 years, range: 0.08 to 1.40 years) before GT insertion for 16 patients tolerating tube placement. The median pre-GT BMI Z-score for these 16 patients was -3.82 (range: -11.89 to 0.90). The median post-GT BMI Z-score from anthropometric data obtained on 17 patients at a median of 2.57 years (range: 0.45 to 5.79 years) after tube placement was -1.17 (range: -11.15 to 1.54). Pre- and post-GT BMI Z-scores were available on 10 patients, and the median change was 0.87 (range: -3.58- 6.72; Wilcoxon signed rank p = 0.32). BMI Z-scores improved or remained stable for seven (70%) patients and decreased in 3 (30%) patients after GT placement. (Figure 3) GT's were not used in two patients as recommended. Caregivers did not administer GT feedings for the patient with a decreased BMI-Z score after GT placement and the second patient chose to not use the GT. Early mortality was not predicted by BMI Z-score alone. Gender, IgA, IgG, and presence of dysphagia were comparable for patients tolerating GT placement and those with early mortality. Minor post-GT complications included wound infection in 1 (4%) patient, tube dislodgement in 1 (4%) patient, and wound leakage in 9 (36%) patients.

**Table 3:** Unadjusted odds ratios and 95% confidence intervals for surviving greater than 30 days after gastrostomy tube (GT) placement by patient characteristics.

	Surviving greater than 30 days after GT placement		
	
Characteristic	Odds Ratio	95% Confidence Interval	P value
Male gender	4.81	0.21 - 319.8	0.47
Age at GT placement, 1 year increase	0.62	0.41 - 0.94	0.02
Dysphagia	0.27	0 - 2.69	0.28
Neurological z-score, 1 point increase	1.02	0.96 - 1.08	0.58
BMI for age, 1 unit increase	1.11	0.78 - 1.57	0.58

Abbr: BMI, body mass index

**Figure 4:**
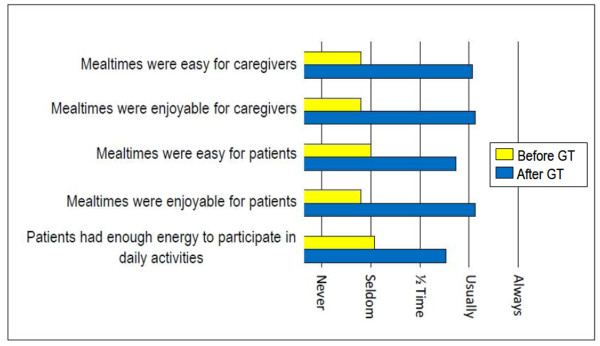
**Mealtime satisfaction and energy to participate in daily activities before and after GT placement**. All pre-post GT comparisons, *p *< 0.0001

All of the 24 (86%) responders to our survey were caregivers of patients who tolerated GT placement and 15 (54%) were caregivers of patients who were alive at the time of this study. Responders were more likely to be caregivers of patients with GT's placed at younger versus older ages (mean age: 12.7 *vs*. 20.2 years, *p = *0.02). Caregivers reported significant improvements in mealtime satisfaction and energy levels for participation in daily activities after GT placement. (Figure 4) Caregivers and patients viewed tube feedings as usually easy to administer and caregivers were almost always satisfied with having the GT's placed.

## Discussion

This retrospective study demonstrates that safe GT placement and caregiver satisfaction can be achieved when patients with A-T have feeding tubes placed at younger ages and before the accumulation of severe complications associated with nutritional compromise or dysphagia with concomitant aspiration. Therefore, we recommend GT's when patients with A-T are young and begin to present with nutrition, respiratory, and dysphagic compromises that are unresponsive to common conservative measures (e.g., dietary modifications and medical therapies) or when feeding disrupts activities of daily living. Although A-T is a rare and complex disease, these findings may have clinical relevance to other children and young adults with neurodegenerative conditions being considered for GT placement.

Survival for patients with A-T has increased with good quality care[10]. Our study was not designed to evaluate the impact of GT's on life expectancy. Longitudinal studies are needed to determine whether early GT placement improves survival. Nonetheless, GT placement is likely to be beneficial for some patients with A-T and we believe that this investigation shows that safety of GT placement can be improved.

At the time of this investigation, our patients who tolerated GT placement had feeding tubes for a median of 5.04 years and approximately two-thirds of them were alive. Those who expired lived to an average age of 20 years, comparable to previously reported survival data[10]. Causes of death were cancer or complications of cancer (55%), congestive heart failure (22%), and respiratory complications including aspiration (22%). Therefore more than half of the deaths among those who tolerated GT insertion were related to conditions or processes unlikely to be influenced by placement of a feeding tube. We continue to follow surviving patients to ascertain the long term impact of GT placement. Given that many factors influence survival we will focus on measures, such as weight gain and stability, frequency of respiratory tract infections, and the onset of dysphagic presentations. Additionally, we will track changes in the ability to participate in daily routines that are most directly related to GT placement. The latter outcome is a key factor in the quality of life of those increased survival years.

Three (11%) patients died within 30 days of GT placement. (Table 1) None of these patients had GT's placed at a tertiary care center. Although our 30-day mortality rate was comparable to the 0 - 27% mortality rates reported in other populations undergoing GT insertion [2,17,21,32,33], the differences between those who tolerated GT placement and those with early mortality are substantial. The three with early mortality had GT's placed at older ages and all demonstrated co-morbidities associated with poor outcomes with GT placement that have been identified in other patient populations with progressive conditions including advanced lung disease, malnutrition, aspiration, and immune deficiency[18,30,32,34-36]. Nonetheless, their immunoglobulin levels, BMI, and neurologic scores did not significantly differ from those who tolerated GT placement. Postponing GT placement as long as possible in patients with A-T may not be in a patient's best interest. Minor complication rates were comparable to the wide range of 6 - 95% previously reported[2,21,32,33,37].

Despite efforts to place GT's at younger ages and before the development of significant co-morbid conditions, some of our patients present to clinic when they are older and have developed risk factors associated with poor outcomes in other patient populations[11-14]. For these patients, we recommend evaluations and interventions that may help minimize complications including nutritional rehabilitation when needed [38-40] and initiation of necessary pulmonary interventions before GT placement[39-41]. Due to the increased risk of complications in all patients with A-T, we recommend placement of GT's at a tertiary medical care center. Additionally, the risk and benefits of GT placement should be discussed with the patient and guardian. Those with more significant lung pathology, including an abnormal chest x-ray, compromised pulmonary function, the need for bronchodilators, older age and concurrent other medical problems may be at higher risk for pulmonary complications during anesthesia[42,43]. Post-operatively, a slow re-introduction of feeds to minimize feeding intolerance, and prompt weaning from mechanical ventilation and early extubation may minimize complications associated with underlying respiratory disease in patients with A-T[44].

Caregiver's were very satisfied with GT's and reported that patients had more energy to participate in daily activities. Our results are comparable to other reports of meals being easier and more enjoyable for caregivers and patients alike[2,23,27]. Nonetheless, one of the limitations of this study was that all respondents were caregivers of patients who tolerated GT placement. Additionally, it is possible that satisfied caregivers were more likely than displeased caregivers to respond to our inquiries and that recollection bias may have played a role in their responses. Another potential limitation is that the survey was comprised of descriptive terms that were not defined precisely and may have been open to variable interpretation.

Many of the limitations of this investigation can be attributed to the retrospective study design and small sample size. For example we were unable to determine the impact of GT feedings on nutritional status because our data did not allow us to standardize the timing of anthropometric measures before and after GT placement, verify whether all patients used GT's as recommended, and obtain complete sets of comparable data for all patients. Additionally the natural course of anthropometric changes associated with disease progression has not been characterized. The small sample size (e.g., three patients in the early mortality group) may contribute to possible discrepancies between findings of clinical and statistical significance. Available records did not permit identification of a control group of patients who did not undergo the procedure despite our recommendations for GT placement. It is notable that GT placement had been recommended for several years prior to their eventual placement in the three patients with early mortality. During the interval between the initial recommendation for GT placement and tube insertion, nutrition and respiratory compromises increased for these three patients. Prospective investigations that track markers of respiratory and nutrition status pre- and post-GT placement are needed to facilitate decision making for determining when GT's should be placed.

## Conclusions

In conclusion, improved safety and easier mealtimes appear to be achievable for A-T patients when GT's are placed at young ages. It is our hope that early GT placement will decrease the severe complications associated with nutritional insufficiency and aspiration secondary to dysphagia. While we are unable to define the optimal age for GT placement, improved outcomes are contingent upon limiting the impact of adverse risk factors generally associated with GT placement as well as those associated with characteristics of the specific disease process. Patients with childhood onset of disorders with predictable progression of the disease process and impaired swallowing may benefit from early versus late placement of feeding tubes.

## Competing interests

The authors declare that they have no competing interests.

## Authors' contributions

MLG participated in the design of the study, data collection, interpretation of the results, and drafted the manuscript. TOC participated in the design of the study, provided and interpreted neurologic score data, and drafted the manuscript. SMM participated in the design of the study, the analysis of data, provided pulmonary input, and drafted the manuscript. KAC participated in the statistical analysis, interpretation of the results, and revised the manuscript, critically. HML participated in the design of the study, provided and interpreted immunologic data, and drafted the manuscript. All authors read and approved the final manuscript.

## Supplementary Material

Additional file 1 **Caregiver Survey**. Click here for file
