# Supplementary material for: Safety and caregiver satisfaction with gastrostomy in patients with Ataxia Telangiectasia
Source: Orphanet J Rare Dis. 2011 May 15;6:23. doi: 10.1186/1750-1172-6-23 (PMC3116459; doi:10.1186/1750-1172-6-23)
Supplement: Additional file 1 — Caregiver Survey. [file 1750-1172-6-23-S1.PDF]

Additional File1: Caregiver Survey

	Never	Seldom	About Half the Time	Usually	Always
Example: I enjoy being outdoors on sunny days					
1. Mealtimes were easy **for me** **before** the G-tube was placed.					
2. Mealtimes were easy **for me** **after** the G-tube was placed.					
3. Mealtimes were enjoyable **for me** **before** the GT was placed.					
4. Mealtimes were enjoyable **for me** **after** the GT was placed.					
5. Mealtimes were easy **for name of pt** **before** the G-tube was placed.					
6. Mealtimes were easy **for name of pt** **after** the G-tube was placed.					
7. Mealtimes were enjoyable **for name of pt** **before** the GT was placed.					
8. Mealtimes were enjoyable **for name of pt** **after** the GT was placed.					
9. Tube feedings are easy **for me**					
10. Tube feedings are easy **for name of pt**					
11. **Name of pt** was too tired or sleepy to participate in most daily activities **before** GT was placed.					
12. **Name of pt** was too tired or sleepy to participate in most daily activities **after** GT was placed.					
13. I think that placing the GT was a good thing to do.					
